# From Biochemical Sensor to Wearable Device: The Key Role of the Conductive Polymer in the Triboelectric Nanogenerator

**DOI:** 10.3390/bios13060604

**Published:** 2023-06-01

**Authors:** Zequan Zhao, Yajun Mi, Yin Lu, Qiliang Zhu, Xia Cao, Ning Wang

**Affiliations:** 1 Center for Green Innovation, School of Mathematics and Physics, University of Science and Technology Beijing, Beijing 100083, China; m202110789@xs.ustb.edu.cn (Z.Z.); d202110423@xs.ustb.edu.cn (Y.M.); b20200369@xs.ustb.edu.cn (Y.L.); d202110424@xs.ustb.edu.cn (Q.Z.); 2 Beijing Institute of Nanoenergy and Nanosystems, Chinese Academy of Sciences, Beijing 100083, China; 3 School of Chemistry and Biological Engineering, University of Science and Technology Beijing, Beijing 100083, China

**Keywords:** biochemical sensor, wearable devices, conductive polymers, triboelectric nanogenerators

## Abstract

Triboelectric nanogenerators (TENGs) have revolutionized energy harvesting and active sensing, holding tremendous potential in personalized healthcare, sustainable diagnoses, and green energy applications. In these scenarios, conductive polymers play a vital role in enhancing the performance of both TENG and TENG-based biosensors, enabling the development of flexible, wearable, and highly sensitive diagnostic devices. This review summarizes the impact of conductive polymers on TENG-based sensors, focusing on their contributions to triboelectric properties, sensitivity, detection limits, and wearability. We discuss various strategies for incorporating conductive polymers into TENG-based biosensors, promoting the creation of innovative and customizable devices tailored for specific healthcare applications. Additionally, we consider the potential of integrating TENG-based sensors with energy storage devices, signal conditioning circuits, and wireless communication modules, ultimately leading to the development of advanced, self-powered diagnostic systems. Finally, we outline the challenges and future directions in developing TENGs that integrate conducting polymers for personalized healthcare, emphasizing the need to improve biocompatibility, stability, and device integration for practical applications.

## 1. Introduction

Since their inception in 2012, triboelectric nanogenerators (TENGs) have revolutionized energy harvesting and active sensing, finding applications in diverse fields such as green energy, molecular detection, healthcare, and gesture recognition [1,2,3,4,5,6]. With their potential to function as both power sources and smart sensors, TENGs offer a promising avenue for sustainable and personalized healthcare solutions. Conductive polymers play a crucial role in the design of TENG-based biosensors and enhancing their applicability in health monitoring, environmental sensing, and point-of-care diagnostics.

TENGs can be seamlessly integrated into intelligent systems for scavenging energy from ambient environments or the human body, providing sustainability, wearability, and portability [7,8,9,10]. In the realm of healthcare, bio-friendly TENGs can be directly worn or implanted into the body to monitor physiological parameters, metabolic status, and even treat diseases with the aid of advanced information technologies. Their simple operation, easy miniaturization, and ability to detect physiological signals make TENGs ideal for clinical devices with predictive, personalized, and participatory characteristics [11,12,13].

Conductive polymer-based TENGs (CPNGs) have shown immense promise in healthcare applications, particularly in connected healthcare and long-term personalized treatments. These innovative devices enable high-quality, real-time monitoring of personal health parameters while providing sustainable and long-lasting power sources [14,15,16]. The integration of CPNGs with conductive polymers enhances their performance by serving as contact layers, amplifying electrical signals, ensuring biocompatibility, and offering flexibility and stretchability. This, in turn, allows for the development of comfortable and unobtrusive wearable devices with improved wearability and user compliance. Furthermore, the functionalization of conductive polymers with specific recognition elements, such as enzymes, antibodies, or aptamers, enables selective and sensitive detection of target analytes in TENG-based biosensors.

However, despite the remarkable advancements in this field, challenges persist concerning power output, device stability, biocompatibility, and integration with other cutting-edge technologies such as flexible electronics and advanced data processing systems [17,18,19,20,21,22,23]. To fully realize the potential of CPNGs in healthcare applications, future research must address these limitations and explore novel strategies for enhancing the performance and capabilities of these versatile devices.

Moreover, we will present the most recent achievements in the field, showcasing the versatility and potential of CPNGs in health monitoring and biochemical sensing. We will discuss various strategies for incorporating conductive polymers into TENG-based biosensors, promoting the creation of innovative and customizable devices tailored for specific healthcare applications.

**Figure 1:**
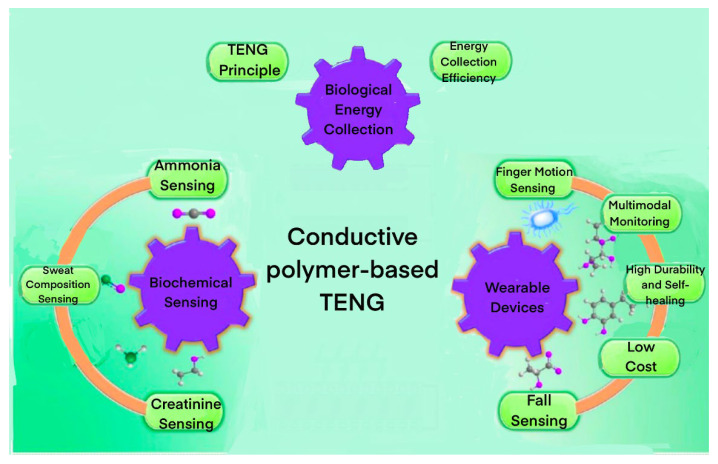
Conductive polymer-based TENG.

In this review, we aim to provide a comprehensive overview of the latest developments and innovations in CPNGs for healthcare applications (Figure 1), with a particular emphasis on the unique contributions of our work. We will discuss the fundamental principles of TENGs, focusing on the integration of conductive polymers and their role in enhancing the performance and utility of TENG-based biosensors. Additionally, we will explore the potential impact of these materials on the future of personalized healthcare and sustainable diagnostic devices.

## 2. CPNG in Biological Energy Collection

### 2.1. The Principle of TENG

TENGs leverage the triboelectric phenomenon and electrostatic induction to convert biomechanical energy into electrical energy [24,25,26,27,28,29,30,31]. When materials with different electronegativities come into contact, electron transfer occurs between them [32,33,34,35,36,37]. As they separate, electrostatic induction prompts electron flow towards the external load, generating an alternating current through repeated contact–separation cycles.

**Figure 2:**
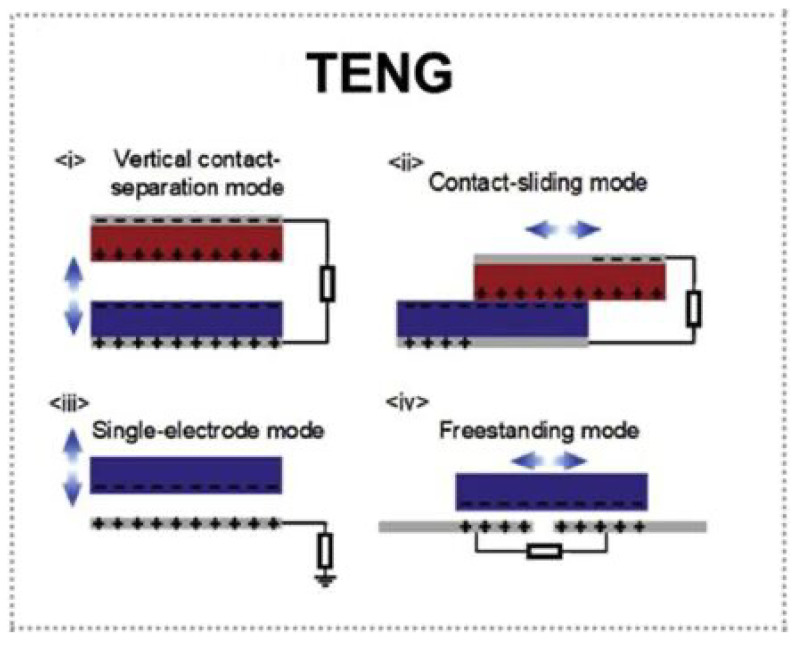
(**i**) Vertical Contact–Separation Mode. (**ii**) Lateral Sliding Mode. (**iii**) Single-Electrode Mode. (**iv**) Freestanding Triboelectric Layer Mode [38]. Elsevier 2020.

TENGs can be categorized into four primary types: the vertical contact–separation mode, lateral sliding mode, single-electrode mode, and free-standing triboelectric layer mode (Figure 2).

Vertical Contact–Separation Mode: In this mode, two triboelectric materials with opposing polarities are positioned closely together. They are periodically brought into contact and separated along a vertical axis. When the materials touch, triboelectric charges generate at their interface. As they separate, the charges redistribute, creating an electric potential difference that drives the electron flow through an external load, producing electric power.

Lateral Sliding Mode: In this mode, two triboelectric materials with opposing polarities slide against each other horizontally. When the materials slide, they generate triboelectric charges at their interface, similar to the vertical contact–separation mode. However, the materials’ relative motion is parallel to their interface, resulting in a continuous change in the overlapping area. This change generates an electric potential difference, which drives the electron flow through an external load.

Single-Electrode Mode: In this mode, only one triboelectric material has an attached electrode, while the other material remains electrically isolated. The isolated material is periodically brought into contact and separated from the material with the attached electrode. Triboelectric charges generated at the interface induce charges on the single electrode during contact and separation. A ground electrode connects to the single electrode through an external load, and the electric potential difference between the ground and the single electrode causes electrons to flow through the load, generating electric power.

Freestanding Triboelectric Layer Mode: In this mode, a freestanding triboelectric layer is sandwiched between two electrodes. The freestanding layer has opposite triboelectric polarities on its two sides. The electrodes are periodically brought into contact and separated from the freestanding layer, causing it to deform. This deformation generates triboelectric charges at the interfaces between the freestanding layer and the electrodes. The resulting electric potential difference between the two electrodes drives the electron flow through an external load. 

### 2.2. Strategies for Improving Energy Collection Efficiency by Introducing Conductive Polymers

Strategies for improving energy collection efficiency by introducing conductive polymers have gained traction in recent years (Table 1). One such strategy involves doping conductive polymers into the friction layer of a TENG, which leads to a better charge transfer, increased surface area, and tunable work function. The integration of conductive polymers with TENGs via the following strategies has expanded their potential applications in various wearable devices and sensors.

#### 2.2.1. Doped into PDMS

**Figure 3:**
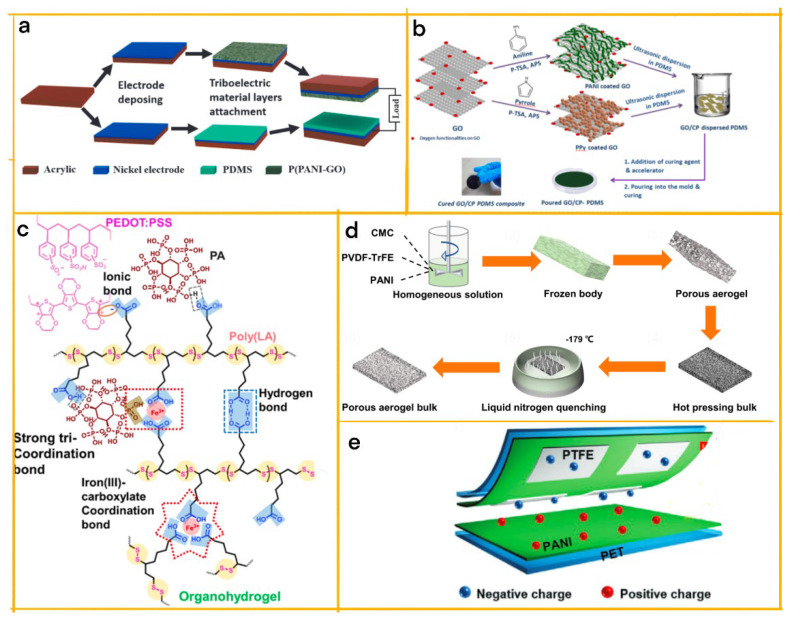
(**a**) Step-by-step fabrication process of the device [39]. 2020 Elsevier. (**b**) Schematic of GO/CP PDMS composites preparation [40]. 2022 RSC. (**c**) Synthetic structure of conductive self-healable organohydrogels (CSOs) as electrodes [41]. 2021 Elsevier. (**d**) Schematic diagram of porous PANI/PVDF-TrFE aerogel bulk preparation process [42]. 2021 Elsevier. (**e**) Structure diagram of the SPTGS [47]. 2021 John Wiley and Sons.

Ahmad et al. developed a novel TENG with an enhanced performance by incorporating conductive polyaniline (PANI) and tribonegative graphene oxide (GO) into the tribopositive material [39]. The unique combination of PANI and GO in the tribopositive layer introduces a new mechanism for performance enhancement, wherein the electron-accepting ability of GO and the conductivity of PANI facilitate the electron flow under an external impact force, thus increasing the surface charge density and overall TENG performance (Figure 3a). The resulting TENG device comprises a polymer with 700 µL PANI and 4 mg mL^−1^ GO as the tribopositive layer, while polydimethylsiloxane (PDMS) serves as the tribonegative layer in a 1 × 2 cm^2^ configuration. This innovative TENG design generates an open-circuit voltage of 314.92 V and a current density of 37.81 mA m^−2^, achieving a peak power density of 10.43 W m^−2^, which is sufficient to power over 175 white light-emitting diodes directly. The proposed tribopositive material, combining PANI and GO, offers a low-cost, easy-to-fabricate solution for creating highly stable and efficient TENGs with a significantly improved performance, paving the way for future developments in sustainable energy harvesting. 

In the study by Jelmy et al., conductive polymers such as polyaniline (PANI) play a significant role in improving the performance of TENG based on polydimethylsiloxane (PDMS) [40]. The researchers incorporated binary hybrids of graphene oxide (GO) and conducting polymers (CPs) such as PANI into the PDMS via an ultrasonication-assisted dispersion technique at room temperature (Figure 3b). The dielectric properties of the PDMS composite were enhanced through various phenomena, such as electronic, vibrational, orientation, ionic, and interfacial polarization. The electron donating–accepting process between PDMS and the GO/CP filler increased the surface charge density of the PDMS composite, boosting the overall TENG performance. The presence of electron-trapping GO in the filler further contributed to the improvement of the composite material’s charge density. The study demonstrated that the PANI nanofiber intercalated GO morphology of the GO/PANI hybrid in the PDMS composite yielded a superior current generation compared to the PPy nanosphere intercalated GO incorporated PDMS system. These findings suggest the potential for utilizing the proposed material in mechanical energy harvesting applications through simple body movements, such as finger tapping and foot stamping. 

#### 2.2.2. Doped into Gel

Another strategy involves incorporating conductive polymers into gels to create more flexible and adaptable TENGs. In a pioneering study by Khan et al. [41]., a fully supramolecular gel-based TENG was developed, incorporating the conductive polymer poly(3,4-ethylenedioxythiophene) poly-styrene sulfonate (PEDOT:PSS) into an electrode gel (Figure 3c). The gel-based TENG, called FSASG-TENG (fully self-healable anti-freezing supramolecular gel-triboelectric nanogenerator), displayed an exceptional performance, maintaining its stability even after 5000 cycles and multiple cut/self-healing processes. The supramolecular gel networks offered remarkable stretchability, reaching up to 50 times strain, and rapid self-healing (4 min for electrode gel and 24 h for tribolayer gel). Notably, the FSASG-TENG showcased a wide working temperature range of −40 to 80 °C, with an energy-harvesting capability verified at these temperatures. Moreover, the energy collected by the gel-based TENG was demonstrated to power commercial electronics, highlighting its potential as a versatile and deformable power source for flexible electronics. This innovative, self-healable, stretchable, and anti-freezing TENG offers a promising solution for energy harvesting in autonomous flexible electronics across a broad temperature range. 

In their research, Yu et al. developed a novel strategy to fabricate a large-scale polyaniline (PANI)/PVDF-TrFE porous aerogel bulk piezoelectric/triboelectric nanogenerator (PTNG) using in-situ doping and liquid nitrogen quenching [42]. The composite aerogel, prepared with a PVDF-TrFE copolymer as the main substrate, sodium carboxymethyl cellulose (SCMC) as a thickener, and PANI as a conductive filler, undergoes rapid cooling with liquid nitrogen after thermoforming to induce the β-phase (Figure 3d). Conductive polymers, such as PANI, play a critical role in enhancing the electrical properties of the composite piezoelectric polymer, allowing for an improved performance. The optimal output of the PANI/PVDF-TrFE PTNG, which contains up to 71% β-phase, achieves 246 V and 122 μA at a frequency of 30 Hz and pressure of 0.31 MPa, with a power density of 6.69 W/m^2^. This innovative strategy facilitates the direct use of the PANI/PVDF-TrFE porous aerogel bulk without the need for subsequent electric field polarization, ultimately reducing energy consumption and shortening the preparation time. Yu et al.’s work addresses the challenge of polarizing PVDF bulk material, paving the way for three-dimensional manufacturing and practical applications of PVDF-based nanogenerators. 

Overall, these strategies highlight the significant potential of conductive polymers for improving energy collection efficiency in TENGs. With continued research and development, these strategies will likely lead to further advancements in sustainable energy harvesting technologies.

## 3. CPNG in Biochemical Sensing

Conductive polymers play a vital role in TENG biochemical sensing applications, such as ammonia sensing and sweat sensing. These polymers, including polyaniline (PANI), polypyrrole (PPy), and poly(3,4-ethylenedioxythiophene) (PEDOT), improve the sensitivity, selectivity, flexibility, biocompatibility, and environmental stability of TENG-based sensors [48] (Table 2). They enhance the sensitivity of sensors by providing efficient charge transport pathways and such polymers can be chemically modified to achieve selective recognition of target analytes [49]. Their inherent flexibility enables the integration with wearable or flexible devices, while their biocompatibility and environmental stability make them suitable for applications involving direct contact with the human body or exposure to challenging conditions. Overall, conductive polymers are essential for the development of advanced, self-powered, wearable, or implantable sensors in TENG-based biochemical sensing applications.

### 3.1. CPNG in Ammonia Sensing

Conductive polymers are vital in TENG ammonia sensing as they enhance the sensitivity, selectivity, and stability of the sensors. For instance, Liu et al. have developed self-powered triboelectric gas sensors that use polyaniline (PANI) film for both the electrode and positive triboelectric layer (Figure 3e) [47]. These sensors measure triboelectric output signal variations without external power and exhibit high sensitivity to low ammonia concentrations due to limited active adsorption sites on the triboelectric layers. Conductive polymer nanofibers and composite materials further improve the TENG performance and expand potential applications, including wearable or flexible devices. 

#### 3.1.1. Conductive Nanofibers

The integration of conductive polymers as nanofibers or conductive composite materials into TENGs not only enhances their performance but also expands their potential applications, such as wearable or flexible TENG devices [50,51,52,53,54,55,56,57]. This is due to the improved mechanical properties, such as flexibility, strength, and durability, which result from creating nanofibers or composite materials.

**Figure 4:**
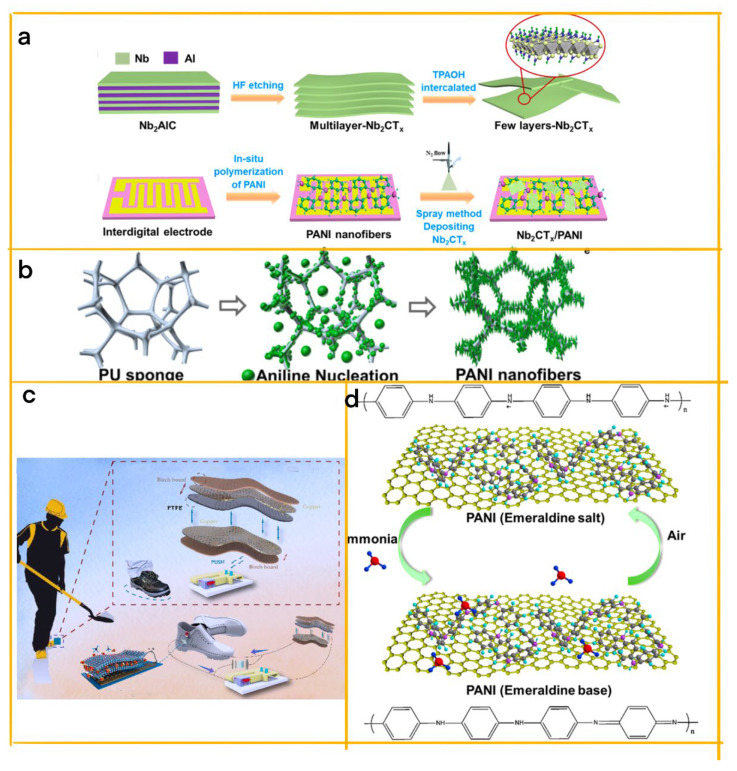
(**a**) Synthesis process of ultrathin 2D Nb_2_CTx nanosheets and fabrication process of Nb_2_CTx/PANI sensor [43]. 2021 Elsevier. (**b**) Schematic illustration of conductive elastic sponge preparation via dilute chemical polymerization method [44]. 2021 Elsevier. (**c**) Self-powered ammonia gas alarm device based on PANI/MXene film sensor for coal miners’ daily shoes [45]. 2021 Elsevier. (**d**) Response mechanism schematic of rGO-PANI nanosheets before and after ammonia gas flow [46]. 2021 ACS.

In a related development, Wang et al. introduced a novel approach to design a selective NH_3_ sensor utilizing polyaniline (PANI) nanofiber-supported Nb2CTx nanosheets, which is directly driven by a TENG at room temperature (Figure 4a) [43]. This unique combination of PANI nanofibers and Nb_2_CTx nanosheets, together with the TENG implementation, enhances the NH_3_-sensing response and provides a broad sensing range of 1–100 ppm NH_3_ at approximately 25 °C under 87.1% relative humidity (RH). This study showcases the potential of integrating TENGs and conductive polymers for the development of high-performance gas sensors.

The high surface area-to-volume ratio of conductive polymer nanofibers is crucial for increasing triboelectric charge generation efficiency. Furthermore, combining conductive polymers with other materials, such as sponge or metal nanoparticles, enables the creation of composites with tunable electrical properties. This, in turn, optimizes TENG performance.

Expanding on these advancements, Liu et al. have designed a conductive and elastic sponge-based triboelectric nanogenerator (ES-TENG) that employs conductive polymers such as polyaniline (PANI) for harvesting random mechanical energy and ammonia sensing [44]. By growing PANI nanowires on the sponge’s surface (Figure 4b), the researchers developed a conductive elastic sponge that can harvest kinetic energy from irregular motion with various amplitudes and directions. The porous sponge and its PANI nanowires, serving as the ES-TENG’s triboelectric layer, offer a large contact area that enhances triboelectric efficiency. Moreover, the conductive PANI coating functions as the ES-TENG’s electrode, generating an output of 540 V and 6 μA. This innovative ES-TENG design demonstrates potential applications in irregular and random mechanical energy harvesting and self-powered NH_3_ sensors, owing to its microporous and nanowire structures, elasticity, conductivity, and ease of fabrication.

#### 3.1.2. Conductive Composite Materials

Conductive polymer composites offer lightweight and cost-effective alternatives to traditional materials, and their compatibility with various fabrication techniques makes them versatile for different processes and substrates [58,59,60,61,62,63]. These properties render conductive polymer nanofibers and composites suitable for a wide range of TENG applications, including energy harvesting, sensing, and actuation.

One notable example is Wang et al.’s innovative self-powered ammonia (NH_3_) sensor, which utilizes polyaniline (PANI)/MXene (V2C) composites as building blocks for a supercapacitor powered by an electromagnetic-triboelectric hybrid generator (Figure 4c) [45]. The MXene’s large accessible surface area enhances the electrochemical activity of PANI, resulting in an improved performance for both the NH_3_ sensor and the supercapacitor. This integrated self-power system demonstrates the potential for creating self-powered gas sensing solutions in industrial and agricultural settings, with applications ranging from mine ammonia leakage alarms to food safety monitoring for remote seniors.

Further building on the potential of conductive polymers, Chang et al. developed a highly sensitive and efficient biosensor based on conductive polyaniline (PANI) and reduced graphene oxide (rGO) nanosheets (Figure 4d) [46]. The PANI-rGO heterostructure exhibits a remarkable sensing performance for ammonia detection, offering linear sensibility, a low limit of detection (46 ppb), and a rapid response time (approximately 75 s). The unique properties of conductive polymers, such as PANI, make them ideal for high-sensitivity sensing materials, while the in-situ growth of monomers on the graphene surface allows for the fabrication of homogeneous PANI-rGO heterostructures without the need for additives.

This innovative, cost-effective, and environmentally friendly biosensor can be integrated with a TENG to create a wearable, self-powered ammonia sensor for early warning systems. The advancements in self-powered sensing systems, as demonstrated by Wang et al. and Chang et al., hold great potential for practical applications and expand the scope of self-powered devices across various industries.

### 3.2. CPNG in Biochemical Sensing

The combination of conductive polymers with TENGs has shown promise in the development of self-powered and highly sensitive sensors for various biochemical sensing applications [64]. The unique properties of various conductive polymers, such as PANI, make them suitable candidates for the development of sensors for detecting various biomarkers, including creatinine or other components in sweat.

#### 3.2.1. Creatinine Sensing

PANI can significantly improve the sensitivity of TENG-based sensors by providing efficient charge transport pathways [65,66,67,68,69,70]. Additionally, PANI can be chemically modified to achieve selective recognition of target analytes. The inherent flexibility of PANI enables the seamless integration of PANI-integrated TENG sensors with wearable or flexible devices, making them suitable for the on-body or non-invasive monitoring of creatinine levels. Furthermore, the biocompatibility of PANI makes it an ideal choice for applications involving direct contact with the human body.

**Figure 5:**
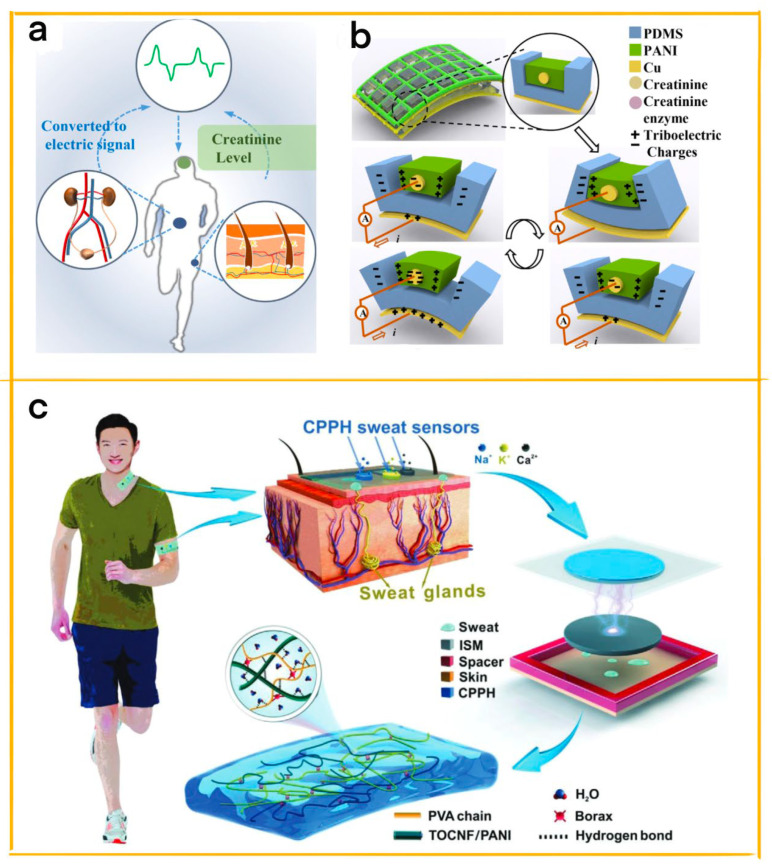
(**a**) General concept of nanosensors for in vivo creatinine level detection through body fluids such as urea and sweat. (**b**) Working mechanism of triboelectric nanogenerator-based nanosensors [71]. 2021 Elsevier. (**c**) Cellulose-based conductive hydrogel for self-powered sweat sensing [72]. 2022 John Wiley and Sons.

In a pioneering study by Luo et al., a cutting-edge, flexible creatinine nanosensor was developed utilizing polyaniline (PANI) and polydimethylsiloxane (PDMS) (Figure 5a,b) [71]. The sensor’s operation relies on the synergy between the TENG and the creatinine enzymatic reaction. The enzyme-modified TENG, composed of PANI and PDMS, exhibits changes in electroconductivity due to the enzymatic reactions. These changes, in turn, affect the triboelectric output and provide information about the ambient creatinine concentration.

**Table 2:** CPNG in Biochemical Sensing.

Date	Sizes	Conductive Polymer	Energy Sources	Outputs	Applications
2021 [71]	2 × 4 cm^2^	PANI	Movement	0.32 nW	Creatinine Sensing
2022 [72]	2 × 1 cm^2^	PANI	Movement	400 V	Sweat Composition Sensing
2020 [73]	2 × 2 cm^2^	PANI	Movement	141 μW	Finger Motion Sensing
2019 [74]	10 × 10 cm^2^	PANI	Movement	200 μA	Finger Motion Sensing
2022 [75]	2 × 2 cm^2^	PPy	Vibration	20.2 V	Fall Sensing
2023 [76]	None	PEDOT:PSS	Vibration	700 V	Fall Sensing

The nanosensor demonstrates remarkable sensitivity at room temperature, with a 51.42% response when the creatinine concentration is 10^−3^ mol/L, and impressive selectivity compared to NaCl, glucose, and urea. Furthermore, the sensor supports a wide range of flexibility in bending angle measurements (10°–40°), making it suitable for wearable sensing applications. Experimental results indicate that this flexible nanosensor enables continuous, non-invasive detection of creatinine, opening new avenues for electronic skin and self-powered healthcare systems.

In conclusion, the unique properties of PANI, such as its flexibility, biocompatibility, and ability to be chemically modified, make it an ideal material for enhancing the performance of TENG-based sensors. The development of wearable, non-invasive sensors such as the one designed by Luo et al. showcases the potential for real-time monitoring of biomarkers such as creatinine. This advancement paves the way for innovative electronic skin and self-powered healthcare systems, revolutionizing personal health tracking and management. As researchers continue to explore the capabilities of PANI, we can expect to see more breakthroughs in the field of non-invasive, real-time health monitoring and personalized medicine.

#### 3.2.2. Sweat Composition Sensing

PANI has the potential to greatly enhance the environmental stability and durability of TENG-based sensors, making them suitable for the long-term monitoring of sweat components under various conditions [77,78,79,80,81,82,83,84]. The unique electrical properties of PANI can be fine-tuned to optimize the performance of these sensors. Moreover, by combining PANI with other materials, researchers can create composites with tailored electrical properties that further enhance the overall performance of the sensor.

In a study conducted by Qin et al., a highly flexible and self-powered biosensor was developed for the real-time analysis and wireless transmission of Na+, K+, and Ca^2+^ levels in sweat (Figure 5c) [72]. This cutting-edge sensor incorporates polyaniline (PANI) as a conductive polymer, which is combined with cellulose nanocomposites to create a hydrogel electrode. The resulting electrode boasts remarkable tensile and electrical self-healing efficiencies of over 95% within 10 s, as well as stretchability up to 1530% and conductivity of 0.6 S/m.

The integration of PANI within the hydrogel electrode greatly enhances the sensor’s performance, enabling the detection of Na+, K+, and Ca^2+^ ions with sensitivities of 0.039, 0.082, and 0.069 mmol^−1^, respectively. By leveraging the triboelectric effect for real-time monitoring, the sensor can wirelessly transmit data to a user interface, providing easy access to the information. The self-powered sweat sensor designed by Qin et al., with PANI as a crucial component, showcases extraordinary flexibility, stability, sensitivity, and selectivity, setting the stage for advanced health monitoring applications.

In conclusion, the unique properties of conductive polymers such as PANI suggest that they can play a significant role in enhancing the performance of TENG-based sensors for various applications. By harnessing the advantages of PANI, researchers can develop highly sensitive, flexible, biocompatible, and stable TENG-based sensors for creatinine or sweat detection. This, in turn, paves the way for the development of wearable, non-invasive, and real-time monitoring of these biomarkers, revolutionizing personal health tracking and management.

In the future, the integration of PANI with other advanced materials and techniques will enable researchers to develop even more sophisticated TENG-based sensors. This will result in expanded applications in various fields such as sports performance monitoring, disease detection and management, and environmental monitoring. The ongoing advancements in PANI-based TENG sensors illustrate the potential for transformative breakthroughs in non-invasive, real-time health monitoring and personalized medicine.

## 4. CPNG in other Wearable Devices 

TENGs that are fabricated with a conductive polymer can present multimodal capabilities, high durability, and low-cost features, which hold great promise for wearable detection applications [85]. The integration of multimodal sensing mechanisms allows these devices to accurately measure and respond to a variety of environmental stimuli, enhancing their versatility in monitoring human movements and other external factors. The improved durability of conductive polymer TENGs ensures a long-lasting, reliable performance even in challenging conditions, making them ideal for wearable technology that may be exposed to various stresses during use [86]. Additionally, the emphasis on cost reduction without sacrificing performance makes these devices more accessible and appealing for widespread adoption in the wearable technology industry. By combining these advantages, conductive polymer TENGs can revolutionize wearable detection applications, enabling the development of advanced, reliable, and affordable sensing solutions for healthcare, sports, and daily life.

### 4.1. Human Motion Sensing

#### 4.1.1. Finger Motion Sensing

Conductive polymer-integrated TENGs offer the potential to create flexible, lightweight, and wearable devices capable of monitoring finger movements. These devices can be incorporated into gloves or attached to the skin to detect bending, stretching, and twisting motions of the fingers. The generated electrical signals can then be processed and analyzed to determine the type and magnitude of the finger movements, opening up applications in various fields, such as virtual reality (VR), gaming, rehabilitation, and human-computer interaction.

**Figure 6:**
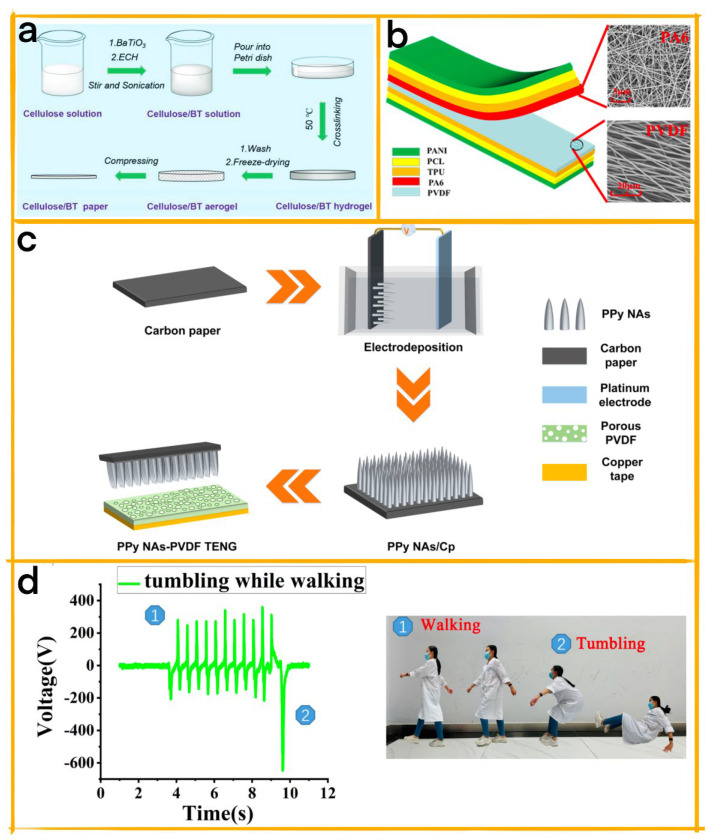
(**a**) Fabrication process of cellulose/BT aerogel paper [73]. 2020 John Wiley and Sons. (**b**) Structural design of wearable TENG and SEM image of PA6 and PVDF nanofiber membrane [74]. 2019 Elsevier. (**c**) Schematic illustration of PPy−PVDF TENG fabrication process: 3D PPyNAs deposited on carbon paper via electrochemical deposition and combined with porous PVDF film [75]. 2022 ACS. (**d**) Walking with a sudden tumble [76]. 2023 Elsevier.

One example is Shi et al.’s innovative biosensor, which uses conductive polymers such as polyaniline (PANI) to enhance the performance of a TENG [73]. By incorporating PANI into the electropolymerization process on a carbon nanotube electrode (Figure 6a), they successfully develop a high-capacitance electrode for supercapacitors. The TENG serves as a sensor unit and operates effectively in a wireless transmission system, enabling the remote monitoring of machine operation and finger movement detection. This research highlights a simple and efficient approach to constructing high-performance TENGs, promoting their application in areas such as wireless transmission and electropolymerization systems.

In a related development, Qiu et al. have designed a wearable TENG that utilizes conductive polymers such as polyaniline (PANI) as electrodes [74]. This TENG, based on ordinary fabrics and integrated with polycaprolactone (PCL) (Figure 6b), ensures a snug fit between the fabric and friction material, enhancing the comfort of wearable, smart health monitoring devices. The TENG exhibits remarkable softness, gas permeability, and flexibility, allowing it to maintain its performance even under various physical manipulations. Additionally, the TENG enables the development of a calibration-free, self-powered sensor for vital sign monitoring and finger tap communication, providing an efficient communication method for patients with language barriers. This wearable TENG demonstrates the potential for long-term reliability within a flexible environment, expanding the applications of conductive polymer-integrated TENGs in wearable devices and health monitoring solutions.

#### 4.1.2. Fall Sensing

Fall detection is crucial for the elderly and individuals with specific medical conditions, as it enables timely assistance and minimizes the risk of injury [87,88,89,90,91,92,93]. Conductive polymer-integrated TENGs can be incorporated into wearable devices such as belts, wristbands, or clothing, allowing for the continuous monitoring of the wearer’s movements. By analyzing the electrical signals generated by the TENGs, the device can differentiate between everyday activities and falls. Upon detecting a fall, alerts can be sent to caregivers or emergency services, ensuring swift intervention.

In a study by Zhang et al., a lightweight wearable TENG with fall detection capabilities was developed using three-dimensional polypyrrole nanoarrays (3D PPy NAs) as conductive polymers (Figure 6c) [75]. Fabricated via electrochemical deposition on carbon paper, various PPy NA morphologies were obtained by adjusting the deposition time. A 1000-s deposition yielded the largest frictional area with poly(vinylidene fluoride) (PVDF) pores. The PPy-PVDF TENG achieved an open-circuit voltage of 20.2 V and a short-circuit current of 1.3 μA. Harvesting mechanical energy from body parts such as the hands and feet, it enabled motion pattern monitoring and sensing, including the detection of falls. Remarkably, the PPy-PVDF TENG illuminated 21 LEDs with a touch and powered wearable watches and portable thermo-hygrometers when integrated with a rectifier circuit and capacitor. This TENG holds potential for wearable devices and self-powered sensing applications, providing enhanced safety features such as fall detection. 

A groundbreaking study by Wang et al. showcases the innovative applications of conductive polymer-integrated TENGs in wearable devices (Figure 6d) [76]. They developed a battery-free human motion sensing system that uses a conductive PVA-PEDOT:PSS hydrogel in a shoe-ground integrated TENG as the signal collection component. This TENG not only serves as a unique energy harvesting method but also offers inventive signal detection capabilities. The conductive PVA-PEDOT:PSS hydrogel effectively captures electrical signals generated by triboelectric friction between the TENG’s friction layers.

To enhance the system’s functionality, the researchers designed an artificial intelligence (AI)-based fall detection system centered around the TENG. They incorporated a custom Bluetooth module to wirelessly transmit collected signals to the cloud, where an anomaly detection AI algorithm identifies fall accidents during walking in real-time and sends instant notifications. This state-of-the-art, battery-free human motion sensing system demonstrates immense potential for wearable electronic devices and serves as a valuable reference for other human motion detection applications.

The TENG system’s unique operating principle functions in a contact-separate mode, capitalizing on the coupling effect of contact electrification and electrostatic induction. The system features friction layers composed of the shoe sole and ground, while the human body and PVA-PEDOT:PSS hydrogel form the TENG electrode, conducting charges generated by friction for electrical signal detection. This innovative approach lays the foundation for a promising future in human motion detection and wearable electronic devices. 

### 4.2. Design Strategy of CPNG-Based Wearable Devices

#### 4.2.1. Multimodal Monitoring

TENG single mode detection, though effective in certain scenarios, suffers from limitations that restrict its overall applicability. The major drawbacks include susceptibility to interference, reduced sensitivity to multiple stimuli, and a narrow scope of environmental conditions (Table 3). These factors can lead to imprecise or unreliable data acquisition, limiting the device’s potential for versatile sensing applications [94]. On the other hand, multimodal detection overcomes these shortcomings by integrating multiple sensing mechanisms, such as the piezoelectric, pyroelectric, and photovoltaic, into a single device. This synergistic approach allows for a more comprehensive understanding of complex environmental stimuli and an improved adaptability across various conditions. Moreover, multimodal detection enhances the reliability and accuracy of data collection, providing a more robust sensing platform for a wide range of applications in fields such as healthcare, energy harvesting, and environmental monitoring. 

The TENG created by Liu et al. combines a conductive polymer film with a magnetic sponge, enabling it to harvest both mechanical and magnetic energy [95]. The device exhibits excellent triboelectric performance, suitable for powering small electronics and wearable sensors. Additionally, the sponge-based TENG generates a significant signal in response to magnetic fields, demonstrating its self-powered sensing capability for external magnetic fields. The TENG also possesses hydrophobicity and lipophilicity, granting it oil–water separation capabilities and potential applications in magneto-driving and target recognition. The TENG-based sensor array showcases sensitive mechanical–magnetic dual-mode responses, accurately mapping external stimuli distributions and integrating with a wireless system for miniaturized portable electronics applications.

**Figure 7:**
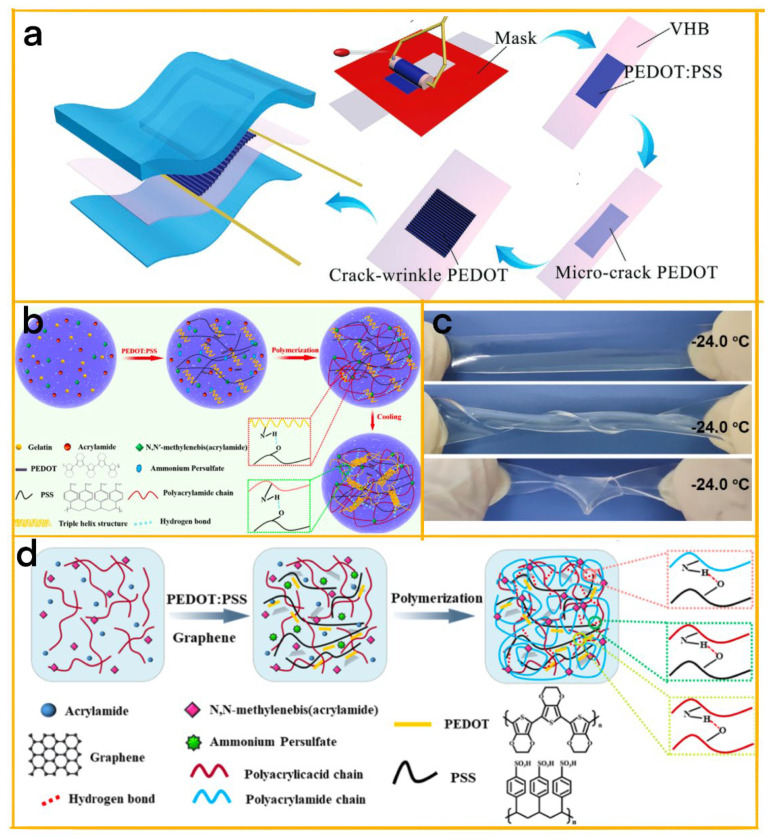
(**a**) Schematic diagram of micro-crack-assisted wrinkled PEDOT:PSS dual-sensor fabrication process [96]. 2022 John Wiley and Sons. (**b**) Schematic illustration of MGP CHs synthetic procedures [97]. 2020 Elsevier. (**c**) Photograph of CNH−3 at 24 °C [58]. 2021 Elsevier. (**d**) Schematic of MAGP hydrogels preparation process [98]. 2022 ACS.

Xiao et al.’s versatile biosensor utilizes micro-crack assisted wrinkled conductive polymers, specifically PEDOT:PSS, to simultaneously detect and differentiate between tensile strain and pressure based on a TENG (Figure 7a) [96]. The wrinkled structure enhances the stretchability of the tensile strain sensor while improving the output and pressure sensitivity of the tactile sensor. By introducing micro-cracks and a cavity, the device’s sensitivity to tensile strain is significantly increased. The device exhibits a gauge factor of 1.75 with 40% linear tensile strain and an exceptional pressure sensitivity of approximately 0.51 V N^−1^ within a 0–24 N pressure range. The device’s stability and durability make it ideal for wearable sensors and self-powered electronics. By adjusting the proportion of wrinkles and micro-cracks, the dual-sensor’s sensitivities can be modulated for versatile applications across various scenarios.

**Table 3:** Design Strategy of CPNG-based Wearable Devices.

Date	Sizes	Conductive Polymer	Energy Sources	Outputs	Applications
2022 [96]	1 × 1 cm^2^	PEDOT:PSS	Movement	20.5 V	Multimodal Monitoring
2020 [97]	None	PEDOT:PSS	Movement	383.8 V	High Durability and Self-healing
2021 [58]	2 × 3 cm^2^	conductive cellulose hydrogels	Vibration	35 V	High Durability and Self-healing
2022 [98]	3 × 1 cm^2^	PEDOT:PSS	Vibration	0.8 μA	High Durability and Self-healing
2019 [99]	2 × 2 cm^2^	PPy	Movement	45 μA	Low Cost
2019 [100]	5 × 2 cm^2^	PPy	Movement	200 V	Low Cost

In summary, both Liu et al.’s TENG and Xiao et al.’s versatile biosensor are excellent examples of multimodal devices with multiple functionalities, such as energy harvesting, sensing, and oil-water separation. These devices showcase the potential of multimodal technologies in the development of wearable sensors, self-powered electronics, and miniaturized portable devices, demonstrating their adaptability and efficiency across different applications and scenarios.

#### 4.2.2. High Durability and Self-Healing

While the innovative approach of TENG to multimodality effectively addresses several limitations, such as susceptibility to interference and reduced sensitivity to multiple stimuli, it is crucial to prioritize the enhancement of durability in these devices. A durable TENG can ensure long-lasting, reliable performance in various environmental conditions and applications, making it a vital aspect of the design process. Although recent developments in multimodal TENGs have shown remarkable progress in stability, further research and optimization efforts should continue to focus on material selection, device fabrication, and integration techniques to prolong the service life of TENGs. This will ultimately result in a more robust and dependable platform for energy harvesting, sensing applications, and self-powered electronics.

Sun et al.’s study utilized a hybrid double network approach combining physically cross-linked gelatin, chemically cross-linked polyacrylamide (PAM), and PEDOT:PSS as a conducting element (Figure 7b) [97]. This resulted in hydrogels characterized by stretchability, conductivity, transparency, and durability. The hydrogels exhibited impressive mechanical properties and self-recovery capabilities due to the physical entanglements and numerous dynamic hydrogen bonds within the double networks. A transparent, wearable strain sensor was fabricated, demonstrating remarkable sensitivity, an ultra-wide sensing range, a short response time, and exceptional durability and reproducibility. Additionally, the hydrogel-based device functions as a highly stretchable TENG (STENG), delivering an efficient energy harvesting performance. The integrated capabilities of strain sensing and energy harvesting make these hydrogels promising for high-performance self-powered wearable devices and stretchable power sources.

Hu et al. have designed flexible, transparent, and conductive cellulose hydrogels for applications in sensors, TENG, and energy harvesters (Figure 7c) [58]. These hydrogels were created through the regeneration of chemically cross-linked cellulose in NaCl aqueous solutions without further treatment. NaCl played a crucial role in determining the hydrogel’s mechanical, optical, conductive, and anti-freezing properties, also contributing to the hydrogel’s safety. Following optimization, the cellulose hydrogel exhibited 94% transparency at 550 nm, 5.2 MPa tensile strength, 235% elongation at break, 4.03 S/m conductivity, and low-temperature tolerance down to −33.5 °C. Moreover, sensors based on the cellulose hydrogel demonstrated a rapid response and consistent sensitivity to tensile strain, compressive pressure, and temperature at both room and sub-zero temperatures, without significant hysteresis. The cellulose hydrogel-based TENG exhibited stability and durability in harsh conditions, while the established method can be used to create flexible, transparent, and conductive cellulose hydrogels with various salts, showcasing universality, simplicity, and sustainability in fabricating cellulose-based flexible conductive devices.

In a study led by Dong et al., a durable and self-repairing dual-network conductive hydrogel was developed, incorporating polyacrylamide (PAAM), poly(acrylic acid) (PAA), graphene (GR), and poly(3,4-ethylenedioxythiophene):poly(styrene sulfonate) (PEDOT:PSS) (Figure 7d) [98]. This unique combination resulted in a hydrogel with adhesive, self-healing, deformable, and conductive properties, demonstrating impressive resilience and longevity. The hydrogel’s dual-cross-linked structure consists of PAAM and PAA, while PEDOT:PSS and GR serve as the conducting components. Its durability and self-repair capabilities are attributed to the effluent gel structure that promotes efficient recovery from damage. A biosensor was designed by embedding the hydrogel between two layers of dielectric carbon nanotubes (CNTs)/poly(dimethylsiloxane) (PDMS), enabling the detection of subtle and vigorous human movements. Furthermore, the durable hydrogel-based sensor functions as a deformable triboelectric nanogenerator (D-TENG), capable of harvesting mechanical energy. The D-TENG exhibits an output voltage and current of 141 V and 0.8 μA, respectively, sufficient enough to power 52 yellow LEDs simultaneously, and small electronic devices such as a hygrometer thermometer.

These studies demonstrate the potential of conductive hydrogels with an enhanced durability and self-healing properties for the development of versatile, self-powered strain sensors and deformable energy sources, paving the way for advancements in wearable devices, health monitoring, and energy harvesting applications.

#### 4.2.3. Low Cost

Although enhancing the durability of TENGs can address certain limitations, such as ensuring reliable performances in various environmental conditions and applications, reducing costs while maintaining a certain level of performance remains a top priority. Developing cost-effective TENGs can enable their widespread adoption across numerous industries and facilitate access to this technology for a broader range of users. To achieve this goal, researchers and engineers should focus on exploring more affordable materials, optimizing fabrication processes, and investigating innovative designs that can provide the desired performance at a lower cost. By striking the right balance between cost reduction and performance, TENG technology can become more accessible, revolutionizing energy harvesting, sensing applications, and self-powered electronics for a wider audience.

Conductive polymers, such as polyaniline (PANI) and polypyrrole (PPy), offer several advantages in the development of high-performance, flexible, and wearable devices, such as TENGs. These polymers are known for their low cost, straightforward synthesis process, and controllable electric conductivity, making them attractive materials for a variety of applications.

**Figure 8:**
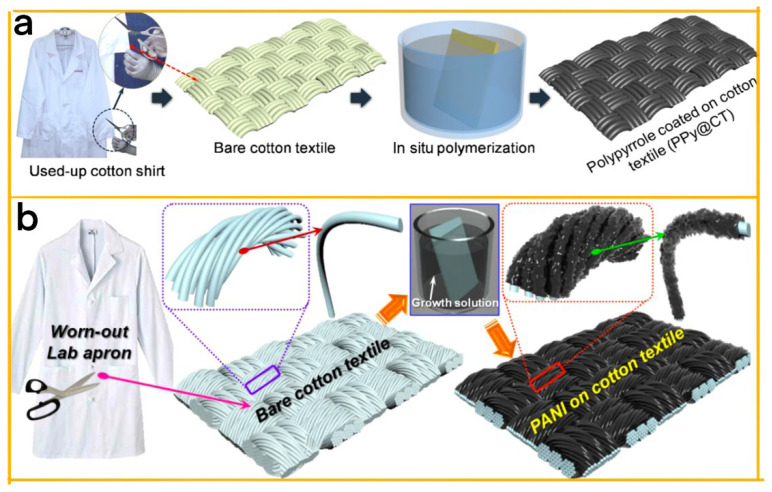
(**a**) PANI deposition process on WCT surface [99]. 2019 Elsevier. (**b**) Polypyrrole-coated cotton textile [100]. 2019 ACS.

In the research conducted by Dudem et al., polyaniline (PANI) serves as a positive triboelectric material and electrode for designing a flexible and wearable TENG (Figure 8a) [99]. The team employed a cotton textile featuring an intertwined micro-fibrous network and good flexibility as a scaffold for depositing PANI using a low-cost, low-temperature, in-situ polymerization method. The fibrous texture of the cotton textile contributes to high surface roughness, further improving the output performance of the TENG. The PANI-coated worn-out cotton textile (PANI@WCT) produced after 20 h of deposition time, yielded an optimal output performance. The electrical stability and mechanical durability of the PANI@WCT-based TENG (PW-TENG) were examined under various long-term cyclic compression operations and mechanical deformation cycles, demonstrating its potential in wearable electronic applications and driving a range of portable electronics. 

Mule et al. have designed a novel, low-cost polypyrrole (PPy)-based flexible and wearable TENG with an outstanding electrical output performance and durability (Figure 8b) [100]. PPy was deposited on a flexible interlaced microfibrous mesh cotton fabric using an in situ chemical polymerization process, creating a PPy-coated cotton textile (PPy@CT) that served as the electrode for the single-electrode-mode TENG. In addition, a sandpaper-assisted microtextured polydimethylsiloxane layer was created on top of the PPy@CT using a straightforward, cost-effective soft-imprint lithography technique, which functioned as the tribo-negative friction layer. The resulting low-cost PPy-based wearable single-electrode-mode TENG (PPy-WSEM-TENG) efficiently converted mechanical energy into electricity through continuous contact and separation with counter friction objects such as dialysis cellulose membranes and human skin (i.e., tribo-positive friction layers). Moreover, the impact of the external pressing force and load resistance on the device’s electrical output performance was analyzed. This affordable PPy-WSEM-TENG not only demonstrated resilience during long-term cyclic operations but also effectively powered portable electronic devices and light-emitting diodes as a self-sustaining energy source. 

## 5. Conclusions and Prospect

Conductive polymers could play a vital and transformative role in the development of both TENGs and TENG-based sensors, enabling efficient energy harvesting from ambient sources and promoting sustainable energy practices and long-term operation. Their inherent flexibility, lightweight nature, and adaptable electrical properties make them ideal candidates for next-generation energy harvesting and biosensing devices. As the demand for clean, renewable energy sources and sustainable sensors continues to grow, further advancements in the synthesis and processing of conductive polymers can yield even more efficient and durable energy harvesters and self-powered sensing systems on the base of TENGs. In the future, we can expect to witness the integration of these remarkable materials into various applications, such as wearable electronics, self-powered sensors, and IoT devices, ultimately revolutionizing our ability to harvest energy from the environment and monitoring the world and ourselves, thus paving the way for a more sustainable future.

### 5.1. Biological Energy Collection

#### 5.1.1. Material Optimization

In the future, researchers can delved into cutting-edge synthesis methods and strategies to create groundbreaking conductive polymers that possess exceptional triboelectric and sensing properties. By exploring the underlying structure–property relationships and fine-tuning the molecular design, researchers can tailor these innovative materials to exhibit enhanced conductivity, mechanical flexibility, and environmental stability. These advances will not only improve the efficiency and reliability of TENGs, but also extend their lifetime, durability, and versatility. Additionally, the development of new conductive polymers may lead to the discovery of materials with unique triboelectric and sensing characteristics, as well as other meaningful properties, such as self-healing or stimuli-responsive behaviors, which could further revolutionize the field of TENGs and expand their application potential.

#### 5.1.2. Device Structure and Surface Modification

To significantly enhance the performance of both TENGs and TENG-based sensors, we can simultaneously focus on developing innovative device architectures and exploring advanced surface modification techniques. By designing new device configurations that maximize charge generation and collection, while maintaining mechanical robustness and flexibility, we can optimize the overall efficiency of TENGs. Additionally, the investigation of advanced surface modification methods, such as patterning, texturing, or functionalization, can further boost the triboelectric and sensing performances by increasing the effective contact area and charge transfer. Combining these efforts can lead to synergistic improvements in the performance of both energy harvesting and environmental sensing, paving the way for more efficient and versatile energy harvesting and sensing devices.

#### 5.1.3. Collecting Energy through Multiple Channels

A researcher can investigate the integration of TENGs with complementary energy harvesting technologies, such as piezoelectric, thermoelectric, or solar generators, to create innovative hybrid systems. By combining the strengths of multiple energy harvesting mechanisms, these hybrid systems can exhibit enhanced efficiency, versatility, and adaptability to varying environmental conditions. This approach can also address the limitations of individual technologies and ensure a more consistent and reliable energy supply, making it suitable for a diverse range of applications, including wearables, IoT devices, and remote sensing. Furthermore, the development of smart energy management systems and advanced materials for hybrid devices can facilitate seamless integration and enable novel energy harvesting solutions that have the potential to revolutionize various industries and contribute to a more sustainable future.

### 5.2. Biochemical Sensing

#### 5.2.1. Improve Sensitivity

To improve the sensitivity of TENG biochemical sensing using conductive polymers, researchers can focus on several key strategies. First, we can investigate new conductive polymer materials or composites that exhibit higher sensitivity to target analytes. This may involve exploring novel materials with unique properties or developing customized composites tailored for specific sensing applications. Second, we can work on enhancing the contact surface area or modifying the surface morphology of the conductive polymers, which can lead to an improved charge transfer and increased sensitivity. Techniques such as nanostructuring or surface functionalization can be employed to achieve these goals. Finally, optimizing the device structure and design is crucial for enabling more efficient charge generation and collection in TENG sensors. This can be achieved by refining the device geometry, incorporating advanced materials, or developing innovative device architectures that enhance the performance of TENG-based biochemical sensors.

#### 5.2.2. Bio-Safety

Enhancing bio-safety in TENG-based biochemical sensing using conductive polymers involves several key approaches. We should prioritize the use of biocompatible and non-toxic materials for TENG-based devices intended for in vivo or in vitro applications, ensuring the safety and compatibility of the sensors with biological systems. Additionally, it is essential to study the long-term stability and degradation of conductive polymers in biological environments, as this knowledge can inform the design of more durable and reliable sensors. Investigations into the effects of various biological factors on the polymers’ performance will contribute to a deeper understanding of their stability. Lastly, developing sterilization methods that do not compromise the sensing performance or stability of TENG-based devices is crucial for their successful integration into medical and biological applications. This may require exploring innovative sterilization techniques or developing sensor designs that can withstand conventional sterilization processes without losing their functionality.

#### 5.2.3. Reduce Interference and Improve Sensing Accuracy

Reducing interference in TENG-based biochemical sensors that use conductive polymers can be achieved through various strategies. Designing TENG-based devices with built-in reference electrodes or compensation circuits can help minimize the effects of environmental factors, leading to more accurate and reliable sensing. Additionally, we can explore the use of selective coatings or molecular recognition elements to improve the sensor’s specificity toward target analytes, which can significantly reduce the impact of interfering substances. These approaches may involve incorporating selective receptors or biomimetic materials into the sensor design. Furthermore, advanced signal processing techniques and machine learning algorithms can be applied to discriminate between target analytes and interfering substances effectively. This approach leverages the power of computational tools to enhance the performance of TENG-based sensors, resulting in more accurate and robust biochemical sensing systems.

### 5.3. Wearable Devices

#### 5.3.1. Personalized Devices

In order to develop personalized TENG-based wearable devices using conductive polymers, researchers should create customizable devices that cater to individual differences, such as body shapes, sizes, and skin types. This ensures a comfortable and effective fit for each user. Additionally, by designing adaptive algorithms that analyze and interpret TENG signals based on each user’s unique physiological and environmental conditions, researchers can create truly personalized wearable devices that respond to an individual’s specific needs.

#### 5.3.2. Durability

Focusing on durability is essential for TENG wearable devices to perform reliably over time. Further studies should be carried out to study the long-term stability of conductive polymers under various conditions, such as mechanical stress, humidity, and temperature. This knowledge will help in selecting materials and designing devices that offer a consistent sensing performance. Furthermore, by exploring self-healing or damage-resistant materials, researchers can enhance the durability and longevity of TENG-based wearable devices, ensuring they remain functional even with daily wear and tear.

#### 5.3.3. Comfortability

The comfortability of wearable devices that are developed on the base of TENG is crucial for user adoption and long-term wearability. We should work on designing lightweight, flexible, and stretchable devices that easily conform to users’ body contours and movements. This will provide a comfortable and unobtrusive experience for the wearer. Additionally, by using biocompatible, breathable, and skin-friendly materials, researchers can minimize irritation and discomfort during extended use, further improving the overall comfort of TENG wearable devices.

## Figures and Tables

**Table 1:** CPNG in Biochemical Sensing.

Date	Sizes	Conductive Polymer	Energy Sources	Outputs	Applications
2020 [39]	1 × 2 cm^2^	PANI	Vibration	37.81 mA m^−2^	Enhance power generation capacity
2022 [40]	None	PANI	Vibration	40 nA	Enhance power generation capacity
2021 [41]	5 × 5 cm^2^	PEDOT:PSS	Vibration	2000 μW m^2^	Enhance power generation capacity
2021 [42]	1 × 2 cm^2^	PEDOT:PSS	Vibration	6.69 W/m^2^	Enhance power generation capacity
2021 [43]	None	PANI	Movement	519 μW	Improve NH_3_ sensing sensitivity
2021 [44]	4 × 4 cm^2^	PANI	Vibration	540 V	Improve NH_3_ sensing performance
2021 [45]	5 × 10 cm^2^	PANI	Movement	500 V	Enhancing the self power capability of NH_3_ sensors
2021 [46]	5 × 5 cm^2^	PANI	Vibration	7.3 μA	Improve NH_3_ sensing sensitivity

## Data Availability

Data available on request from the authors.
